# Comparison of a new nanoform of the photosensitizer chlorin e6, based on plant phospholipids, with its free form

**DOI:** 10.1002/2211-5463.12359

**Published:** 2018-01-02

**Authors:** Lyubov V. Kostryukova, Vladimir N. Prozorovskiy, Natalya V. Medvedeva, Olga M. Ipatova

**Affiliations:** ^1^ Institute of Biomedical Chemistry Moscow Russia

**Keywords:** accumulation in tumor, chlorin e6, drug delivery, lipid nanoparticles, oncology, photodynamic therapy

## Abstract

Photodynamic therapy is an advanced method of treating cancer and various benign diseases, including infections. It uses light‐activated molecules [photosensitizers (PSs)] to generate reactive oxygen species (ROS) when irradiated with light of a specific wavelength. This study examined the photophysical and photosensitizing activity of the PS chlorin e6 incorporated in a delivery system based on plant phospholipids. This new nanoform of chlorin e6 comprised particles with a diameter of 18.4 ± 2.5 nm and zeta potential of −34.6 ± 3.0 mV. Incorporation of chlorin e6 in phospholipid nanoparticles was observed to cause a bathochromic shift of Q‐band absorption maximum by 14 nm without an absorption change in the range of the Soret band. Fluorescence intensity of chlorin e6 embedded in the phospholipid nanoparticles increased 1.7‐fold. Chlorin e6 in phospholipid nanoparticles, when irradiated, was able to generate ROS as shown by oxidation of polyunsaturated fatty acids of the phospholipid matrix of the delivery system and reduced l‐glutathione. *In vivo* it was demonstrated that the new nanoform of chlorin e6 provides more accumulation of PSs in tumor tissue than its free form. Moreover, its accumulation in the skin was lower and its elimination from the skin almost five times faster than when administered in free form. The observed differences of this new nanoform of chlorin e6 should lead to enhancement of antitumor efficacy and a decrease in phototoxicity.

**Abbreviations**
Ce6: chlorin e6
FAME: fatty acid methyl ester
GSH: l‐glutathione reduced
LC‐MS: liquid chromatography–mass spectrometry
PC: phospholipids, phosphatidylcholine
PDT: photodynamic therapy
PS: photosensitizer
PUFA: polyunsaturated fatty acid
ROS: reactive oxygen species
TFA: trifluoroacetic acid

Photodynamic therapy (PDT) is an advanced method of treating cancer and various benign diseases, including infections 1. The main components for photochemical reactions are light‐activatable molecules [photosensitizers (PSs)], light and oxygen 2. The principle of the method of PDT is that PSs – under irradiation by light of a specific wavelength – are able to initiate generation of singlet oxygen and other reactive oxygen species (ROS) that either kill tumor cells directly, or damage tumor vasculature 3, 4. PDT has a number of advantages over conventional methods of cancer treatment: (a) local impact, (b) non‐invasiveness and efficacy, especially in surgically untreatable cases, (c) no long‐term adverse events compared with chemotherapy, and (d) absence of systemic immunosuppression, as opposed to chemotherapy and ionizing radiation 5. However, adverse events and significant phototoxicity because of non‐specific distribution in the body have encouraged researchers to develop new PSs. Of all the PSs of natural or synthetic origin, the most promising is chlorin e6 (Ce6), boasting high efficacy and very low toxicity 6. There are a number of drugs based on Ce6 or its derivatives that have been already created and applied in the clinic, such as Photolon, Radachlorin, Photoditazin, Taloporfin and Pirlitin.

Ce6 and its derivatives have maximum absorption in the ranges of 400–410 and 650–670 nm 7, 8. The photodynamic activity of Ce6 is revealed when exposed to light in the range 650–670 nm, which corresponds to the range of optical transparency for non‐pigmented tissues. Light within this spectral range may penetrate as deep as 7 mm 9. Due to intensive fluorescence, Ce6 and its derivatives may be used in neoplasm photodiagnostics 9. However, just like many other PSs, Ce6 is characterized by low solubility in water, which substantially restricts its medical use 10. PS disadvantages also include significant phototoxicity, involving decrease in healthy cell resistance to ROS generated during irradiation. To avoid adverse events, the patient has to adhere to a strict light regime after a PDT session. The duration of the regime depends mostly on the PS type and the dose used.

Considering Ce6 efficacy, safety, and high potential in the area of theranostics (therapy and diagnostics) 1, many researchers are seeking approaches to improve its characteristics, such as its solubility, phototoxicity and accumulation in tumors. One of these approaches, which has proved successful in recent years, is supplying the drug with a delivery system. Development of nanotechnologies in the past years has initiated the extension of studies focused on the creation of effective drug delivery systems. The introduction of new materials and technologies enables the design of transport systems using various nanoparticles. For example, several delivery systems have been developed for Ce6 based on polymers 11, 12, gold nanoparticles 13, carbon nanotubes 14, silicon nanoparticles 15, liposomes 10, 16, 17, etc. Liposomes are colloidal particles used to deliver drugs, including PSs. As opposed to the other above‐mentioned delivery systems, they feature properties such as biocompatibility, biodegradability, low toxicity, and variability of structure and physico‐chemical properties 10, 18. Thus, varying the lipid composition allows particles of different sizes to be obtained, to change their charge, and to control the release of an encapsulated drug; the surface of liposomes may be easily modified using functional ligands to improve targeting, and so on 19.

Analysis of the literature shows that using liposomes as delivery systems for PSs protect them from inactivation in the plasma during blood circulation, facilitates their accumulation in the tumor tissue and increases photocytotoxicity, all of which improves PDT efficacy. For example, Yen *et al*. 20 report growth of PDT efficacy and increased accumulation of di‐*N*‐methylglucamine salt of Ce6 in tumors when included in liposomes based on egg phosphatidylcholine, cholesterol and poly(ethylene glycol) with size of 178 ± 10 nm. Similarly, Namiki *et al*. 21 used Ce6 sodium salt (Ce6‐Na) as an example to show that Ce6, when included in stealth liposomes of 100 nm in diameter, had substantially better penetration into cells *in vitro* and efficiently inhibited tumor growth *in vivo*. It should be noted that the size of liposomes is an important factor affecting PS accumulation in tumors 21, 22, 23.

We have developed a drug delivery nanosystem based on soya phospholipids with particles less than 20 nm in size. A method allowing for the incorporation of drug substances from various therapeutic groups into these nanoparticles was also developed 17. The studies demonstrated that incorporation of a drug in the phospholipid nanoparticles of that size substantially affects its pharmacokinetics. For example, it certainly changes drug distribution over blood components, increases accumulation in areas of inflammation, significantly enhances bioavailability, and consequently improves efficacy of its therapeutic effect 24, 25, 26, 27. The approaches developed by us were used to obtain a composition of Ce6 incorporated in phospholipid nanoparticles (Ce6–PC) of extremely small size (up to 30 nm) as a transport system. The results from examining the physico‐chemical properties and photosensibilizing activity of Ce6–PC are presented in this paper.

## Materials and methods

### Materials

Active substance of Ce6 di‐*N*‐methylglucaminate (Ce6) was obtained by Ivanovo State University of Chemistry and Technology (Russia). The following reagents were used: Lipoid S100 (Lipoid, Ludwigshafen, Germany), maltose (Merck, Darmstadt, Germany), phosphate‐buffered saline (PBS; Sigma‐Aldrich, St Louis, MO, USA), l‐glutathione (GSH) reduced (AppliChem, Darmstadt, Germany), methanol – HPLC grade (Fisher Scientific UK Ltd, Loughborough, UK), trifluoroacetic acid (TFA) (Fluka Chemie AG, Buchs, Switzerland). All solutions were prepared using distilled water. The Moscow Regional Blood Transfusion Center provided blood plasma of healthy volunteers.

### Ce6–PC nanovesicle preparation and characterization

Nanovesicles of Ce6–PC were prepared by homogenization using microfluidizer M110EH30K (Microfluidics, Westwood, MA, USA). Crude dispersion was prepared by intense mechanical stirring (in a vortex mixer) of Lipoid S100 (50 mg·mL^−1^) and Ce6 (5 mg·mL^−1^) in water heated to 45 °C and homogenized at a pressure of 1000 atm and temperature of 45 °C. After adjusting the pH to 7.0, the ultra‐fine emulsion was filtered with a YY3009000 system (Millipore Corp., Billerica, MA, USA) with 220‐nm filter pores. The efficacy of Ce6 incorporation in the phospholipid particles was determined using ultrafiltration 28.

The size of Ce6–PC particles was determined using dynamic light scattering on a Zetasizer Nano series Nano‐ZS (Malvern, UK) with Malvern zetasizer 6.20 software. The hydrodynamic diameter of the particles was measured by dynamic He–Ne laser (633 nm, 4 mW) light scattering at an angle of 173° at 23 °C. Zeta potentials were determined by laser Doppler micro‐electrophoresis at an angle of 13°.

Absorption spectra were recorded on an Agilent 8453 spectrophotometer (Agilent Technologies, Santa Clara, CA, USA) with HP uv visible chemstation software; fluorescence spectra were registered using Varian Cary Eclipse fluorescence spectrophotometer (Agilent Technologies) with cary winuv software.

### Liquid chromatography–mass spectrometry analysis of reduced l‐glutathione

Decrease of concentration of the reduced form of GSH in the presence of PS on irradiation was carried out in 0.1 m PBS (pH 7.4) and in the blood plasma of a healthy donor. Solutions of Ce6, Ce6–PC and GSH were prepared in distilled water with a concentration of 5.5 mg·mL^−1^ for PS and 1 mg·mL^−1^ for GSH.

Of one of the two aforementioned media, 800 μL was mixed with 100 μL of GSH solution and 100 μL of PS solution. In the control samples, PS was replaced by 100 μL of water. After 15 min dark adaptation, the samples were irradiated by light with a wavelength of 650 nm and power of 300 mW using Laser Pointer (Shezhen, China) positioned vertically at 4 cm above the irradiated sample. After 10 min of irradiation, 100 μL portions were sampled to analyze GSH content.

Of new samples, 100 μL was mixed with 400 μL of methanol, stirred using an orbital shaker (IKA MS3 basic; IKA, Staufen, Germany) for 3–5 min, and centrifuged to precipitate proteins on the MiniSpin plus (Eppendorf, Hamburg, Germany) centrifuge at 14 000 ***g*** for 10 min. Supernatant was diluted 10 times by 0.1% TFA solution and analyzed for GSH content.

Mass spectra were acquired using a high‐performance liquid chromatograph (Agilent 1200), coupled to a quadrupole mass spectrometer detector (Quadrupole LC/MS 6130; Agilent Technology) with an electrospray ionization source. The analysis was performed using selected ion monitoring in positive mode. Ten microliters of sample was injected onto a 4.6 × 150 mm (5 μm) Eclipse XDB‐C18 column (Agilent Technology) and eluted with a mobile phase consisting of methanol/water, 5 : 95 (v/v) with 0.1% TFA with a run time of 10 min. Column temperature (25 °C) and the eluent flow rate (0.5 mL·min^−1^) were maintained at constant levels. GSH was registered at *m*/*z* of 308. Chromatograms were treated using ChemStation B.01.03 (Agilent Technology, Santa Clara, CA, USA) software.

An aqueous stock solution of GSH (1 mg·mL^−1^) was used for preparation of standard solutions with final concentrations of GSH of 10 000, 5000, 1000, and 100 ng·mL^−1^; these solutions were prepared by sequential dilution of the stock solution with water. A calibration plot of the ratio of peak areas of GSH concentration in this range was linear. The correlation coefficient was 0.99998.

### Liquid chromatography methods for UV detection of Ce6

Ce6 was analyzed using a high‐performance liquid chromatograph (Agilent 1100) coupled to UV detection. Ce6 was registered at λ = 400 nm. The sample was injected (10 μL volume) onto a 4.6 × 150 mm (5 μm) Eclipse XDB‐C18 column (Agilent Technologies) and eluted with a mobile phase consisting of acetonitrile with 0.1% TFA (B)/water with 0.1% TFA (A). The column was pre‐equilibrated at 50% B. After 0.2 min a linear gradient of 13.3% B per minute for 3 min was implemented. The column was then washed with 90% B for 7 min and subsequently re‐equilibrated at 50% B for 3 min, resulting in a total run time of 13 min. Time retention in this condition for Ce6 was 5.74 min.

Calibration dependence of the ratio of peak areas was linear in the Ce6 concentration range from 5 to 50 μg·mL^−1^. The correlation coefficient was 0.99998. Chromatograms were treated using chemstation for lc a.09.03 software.

### Liquid chromatography methods for mass spectrometry detection of Ce6

MS detection was performed using an Agilent 1200 coupled to a quadrupole mass spectrometer detector Quadrupole LC/MS 6130 (Agilent Technologies) with an electrospray ionization source. The analysis was performed using selected ion monitoring in positive mode. Ce6 was registered at *m*/*z* of 597.7. The mobile phase consisted of acetonitrile with 0.1% formic acid (B)/water with 0.1% formic acid (A). The column and chromatographic conditions for separation of Ce6 were the same as in the LC methods for UV detection. Calibration dependence of the ratio of peak areas was linear in the Ce6 concentration range from 0.1 to 10 μg·mL^−1^. The correlation coefficient was 0.9995. Chromatograms were treated using chemstation b.01.03 software.

### Fluorescence spectroscopy for detection of photosensitizer accumulation and clearance from animal skin

Studies of the dynamics of PS accumulation and clearance from skin were by fluorescence spectroscopy, assuming the fluorescence intensity is proportional to its concentration, using a fiberoptic spectranalyzer LESA‐01‐Biospec (Biospec, Moscow, Russia) 29. Fluorescence of the PS was excited at 632.8 nm by a He–Ne laser, with the intensity of fluorescence characterized by an integral area under the obtained fluorescence spectra in the spectral range of 650–700 nm, normalized to the integral intensity of scattered excitation light. Intensity of fluorescence in the tissue of animals of control groups was evaluated the same way. The data of values of fluorescence in skin samples at different time points after intravenous administration was used for evaluation of dynamics of PS accumulation upon administration in both injected forms.

### Animals and tumor models

Male BDF1(C57Bl6*DBA\2) mice weighing 23 ± 3 g were inoculated subcutaneously with 10^6^ Lewis lung carcinoma cells suspended in 300 mL 199 medium in the thigh muscle from the outer side. After 8 days from inoculation, the animals were divided into two groups (*n *=* *30 per group). Free Ce6 or Ce6–PC nanoparticles were intravenously administrated into mice at a Ce6 dose of 5 mg·kg^−1^. At certain time intervals, three animals from each group were sacrificed with an ether overdose. Tumor tissue and pieces of skin were excised and analyzed using liquid chromatography–mass spectrometry (LC‐MS) and contact fluorescence methods.

All animal experiments were performed in accordance with The National Standard of Russia 33044‐2014 ‘Principles of good laboratory practice’.

### Tumor tissue

Extraction of Ce6 required homogenization of tissue. Briefly, 300 μL of water was added to the weighed tumor tissue samples (100 mg) and homogenized using a supersonic Sonorex digital 10P (Bandelin, Berlin, Germany). Then, 450 μL ice‐cold methanol was added to 50 μL of homogenate and after centrifugation (3000 *g*, 4 °C, 15 min) the supernatant was analyzed for Ce6 content as described in ‘Liquid chromatography methods for mass spectrometry detection of Ce6’. It was shown in control experiments that the recovery of Ce6 was not below 80%.

### Gas chromatographic analysis of fatty acid methyl esters

The HCl‐catalyzed procedure of transesterification of fatty acids was modified from that of 30. Fatty acid methyl esters (FAMEs) were prepared by incubation Ce6‐PC in methanol (20 μL, 5 mg·mL^−1^) for 1.5 h with 3.6% HCl in dry methanol (1100 μL) at 80 °C. The resulting FAMEs were extracted with 2.2 mL hexane with octacozane as the internal standard (10 μg·mL^−1^) at room temperature for at least 1 h. The extract was analyzed in hexane by gas chromatography using an Agilent Technologies 6890 N gas chromatograph, fitted with a 7683B series injector, a mass spectrometer (model 5973) and a 30 m × 0.25 mm i.d., 0.25 μm film thickness capillary column (HP‐INNOWAX 19091N‐133; Agilent Technologies). Helium was the carrier gas at a flow rate of 1 mL·min^−1^. The column temperature was held at 100 °C for 2 min, then was temperature‐programmed to 240 °C at 15 °C·min^−1^, which was maintained for a further 20 min. FAMEs were identified using MSD ChemStation E.02.00.493 with the National Institute of Standards and Technology Mass Spectral Search Program/NIH Mass Spectral Library. The amount of each FAME was calculated using octacozane as the internal standard.

## Results and Discussion

### Preparation and characterization of nanoform photosensitizer on a base of Ce6 and plant phospholipids

Chlorin e6 is characterized by low water solubility, which hampers its clinical use 10, 31. Taking into account its physico‐chemical properties and its high photodynamic efficacy, water‐soluble derivatives of Ce6 were created 31. For example, a drug named Taloporfin was developed in USA based on mono‐l‐aspartyl chlorin (NPe6). Another drug, Photoditazin, was developed and is clinically used in Russia, having di‐*N*‐methylglucamine salt of Ce6 as the active ingredient. However, medical use of these drugs has revealed a number of adverse events, such as: (a) unstable spectral characteristics, due to a soluble form of the drug; (b) limited shelf life; and (c) prominent phototoxicity, caused by low clearance rate. For these reasons, a search was initiated for approaches to create new Ce6‐based drug forms with high stability, high accumulation efficacy and low phototoxicity. For example, in Ref. 32 it was shown that efficacy of Ce6 embedding into liposomes of 100 nm in diameter based on soya phosphatidylcholine correlated with their hydrophobicity. In Ref. 20 the results were presented of studying Ce6 accumulation in tumors, and the enhancement of PDT efficacy of Ce6 incorporated in the liposomal particles based on egg phosphatidylcholine and poly(ethylene glycol) of about 200 nm in size was noted. In general, a consensus in literature exists that decreasing carrier size will improve tumor deposition 33. Thus, in Ref. 34 it was shown that a twofold increase in the size of particles transporting PS reduces the efficiency of PDT almost 4‐fold. Given this a phospholipid transport system with an extremely small particle size (20 nm), developed by us 17, was used to prepare a Ce6–PC composition with a high efficiency.

**Figure 1:**
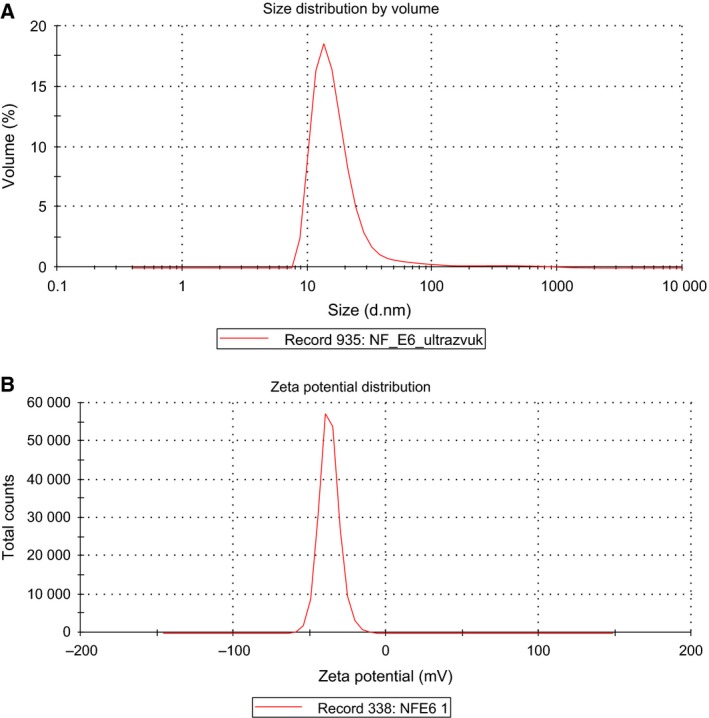
Size distribution (A) and zeta potential (B) of Ce6–PC nanoparticles.

Figure 1 presents data related to determining the particle size and zeta potential of the composition obtained based on the di‐*N*‐methylglucamine salt of Ce6 and phosphatidylcholine using dynamic light scattering. Figure 1A shows that the average size of nanoparticles of the main fraction (≅ 98%) is 18.4 ± 2.5 nm. Figure 1B illustrates a typical distribution of Ce6–PC particles by charge. Average zeta potential is ‐34.6 ± 3.0 mV. Zeta potential, a characteristic of surface charge, determines anti‐aggregation stability of colloid systems 35, 36. The obtained value of zeta potential indicate the stability of Ce6–PC particles in colloidal solution. It is important to note that ≥ 95% of Ce6 is embedded in phospholipid nanoparticles as shown with the ultrafiltration method.

### Spectral and fluorescence characteristics of Ce6 in phospholipid nanoparticles

**Figure 2:**
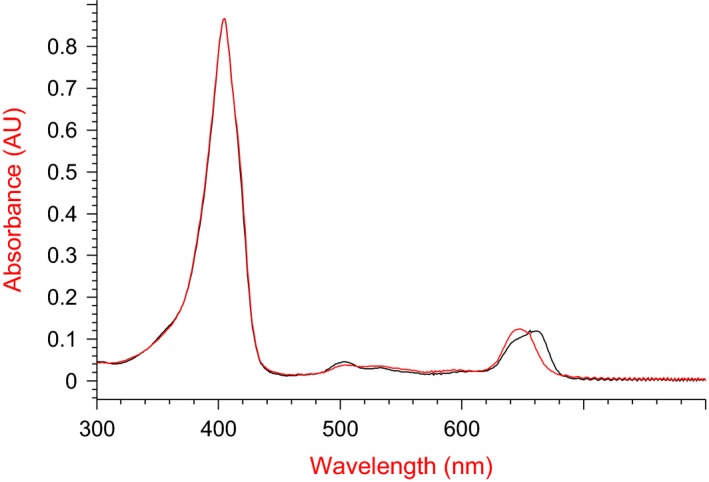
Absorption spectra of di‐*N*‐methylglucamine salt of Ce6 (red curve) and Ce6–PC (black curve) in PBS (pH 7.4). Concentration of Ce6, 5 μg·mL ^−1^.

PS efficacy is defined by its spectral characteristics: (a) light absorption in the spectral range of biotissue transparency (red and infrared ranges), and (b) fluorescence ability. It is known that the spectral properties of chromophores depend on polarity of the environment 37. It was earlier shown that embedding hydrophobic Ce6 into liposomes is largely limited to the outer surface of the phospholipid bilayer 38. The Ce6 molecule were somewhat submerged in the bilayer, settling down between the polar and hydrophobic areas of phospholipids. Thus, surrounding Ce6 with phospholipid molecules may influence its spectral characteristics. We investigated the absorption spectra of Ce6 incorporated in Ce6–PC nanoparticles. Figure 2 illustrates the absorption spectra of the di‐*N*‐methylglucamine salt of Ce6 as a free substance and within phospholipid nanoparticles.

According to Fig. 2, absorption near 400 nm (Soret band) typical of porphyrins didn't change for Ce6 embedded into phospholipid nanoparticles. The Q‐band (λ = 650 nm) presents a bathochromic shift: the absorption spectrum is shifted toward the long‐wave region by 14 nm. It should be noted that the red shift of the Ce6–PC spectrum enables enhancement of the penetration depth of irradiation light, to reduce the absorption by the blood pigments, and consequently to improve PDT efficacy 39, 40.

**Figure 3:**
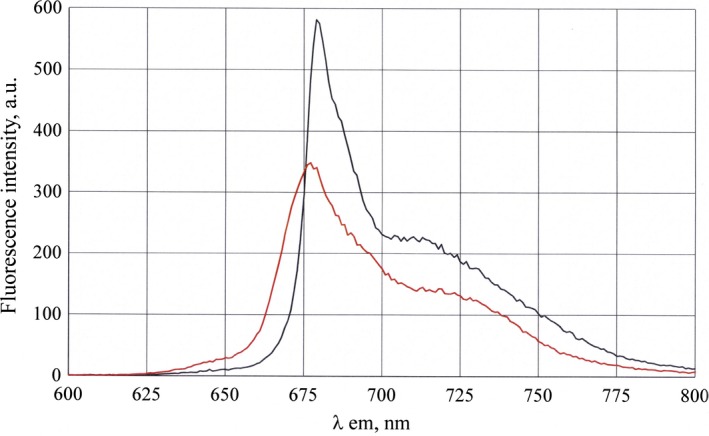
Fluorescence spectra of Ce6 (red curve, λ_max _= 677 nm, fluorescence intensity (FI) = 345) and Ce6 embedded into phospholipid nanoparticles (black curve, λ_max_ = 680 nm, FI = 575) in PBS (pH 7.4). Excitation wavelength λ_ex_ = 675 nm.

As noted above, fluorescence is one of the important characteristics of Ce6. We have studied the impact of incorporating Ce6 in the phospholipid nanoparticles based on its fluorescence properties. Figure 3 presents fluorescence spectra of Ce6 in free form and within phospholipid nanoparticles at an excitation wavelength of 675 nm. The fluorescence spectrum for Ce6–PC has λ_max _= 680 nm *vs* 677 nm for Ce6. The presented data show that the fluorescence intensity of Ce6 as a part of Ce6–PC is 1.7 times higher. It should be noted that enhancing fluorescence intensity of Ce6 due to embedding into phospholipid nanoparticles expands the diagnostic opportunities for Ce6 application.

Therefore, inclusion of Ce6 into phospholipid matrix enables enhancement of its spectral characteristics as a photosensitizing agent for PDT, with observed red shift of Q‐band absorption maximum and increased fluorescence intensity as compared with free Ce6.

### Photodynamic activity of Ce6 embedded in phospholipid nanoparticles *in vitro*

The efficacy of PSs is defined by their ability to generate ROS (type I reaction) or singlet oxygen (type II reaction) when exposed to the light. Balance between the two processes depends on PS type, oxygen concentration and, for the type I reaction, substrate affinity to PS 10. Singlet oxygen and ROS have high reactivity. Due to their short life cycle, their action is of a local nature.

Soybean phospholipids included in Ce6–PC are characterized by a high content of polyunsaturated fatty acids (PUFAs), which can become free radical scavengers. Taking this into consideration, we have evaluated how PUFAs contained in phosphatidylcholine comprising the matrix of Ce6–PC are oxidized under irradiation (Table 1). As expected, Ce6 under irradiation becomes a source of ROS, activating PUFA oxidation. As seen from the Table 1, the content of oleic, linoleic and linolenic fatty acids in the samples decrease by 16%, 29% and 37%, respectively.

**Table 1:** Content of polyunsaturated fatty acids in the Ce6–PC samples before and after irradiation by laser at λ = 650 nm. Values are shown in relative units – the ratio of the peak area of the polyunsaturated fatty acid to that of the internal standard. *P ≤* *0.05; **P ≤* *0.01

Sample	Oleic	Linoleic	Linolenic
Ce6–PC before irradiation	0.40 ± 0.03	1.7 ± 0.1	0.113 ± 0.009
Ce6–PC after irradiation	0.33 ± 0.03*	1.213 ± 0.006**	0.071 ± 0.003**

**Figure 4:**
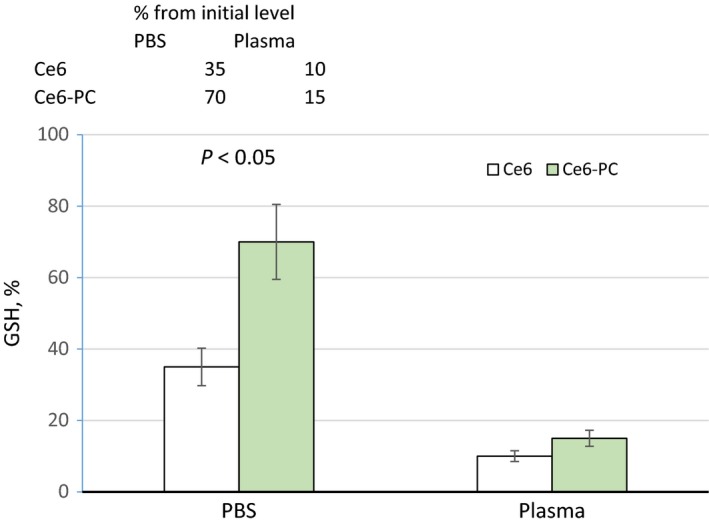
Alteration of GSH content (as percentage of pre‐irradiation GSH level) in PBS and blood plasma after 10 min irradiation of the samples containing Ce6 and Ce6–PC by laser light at λ = 650 nm.

The photoactivity of Ce6 and Ce6–PC was also studied by the ability to oxidize GSH. The GSH redox system is the primary cellular antioxidant system, controlling the cell's ability to withstand oxidative stress – shift in prooxidant/antioxidant balance 41, 42. Therefore, given the fact that Ce6 under irradiation becomes a free radical source, the cellular redox system under *in vitro* conditions reacts to the generation of ROS by the degree of GSH oxidation. We have conducted a comparative study of the oxidation degree of GSH in the presence of Ce6 and Ce6–PC within PBS and blood plasma of a healthy donor. Figure 4 illustrates the results of measuring GSH concentration in the medium under study after 10 min sample irradiation. It is important to note that GSH concentration did not decrease in the samples not exposed to irradiation.

As seen from the data presented in Fig. 4, GSH oxidation in PBS is more effective in the presence of free Ce6 (*P *≤* *0.05). This is due to the fact that in the samples containing Ce6–PC, ROS is spent on oxidation not only of GSH, but also, as shown above, on the oxidation of unsaturated fatty acids of PC. In plasma, the results of GSH oxidation by irradiation of free Ce6 are practically the same as with the phospholipid composition. This is because in the plasma, the oxidation is also of the PUFAs of phospholipids of lipoproteins. Oxidation of PUFA under the action of ROS initiates lipid peroxidation. The free radicals formed as a result of lipid peroxidation also cause oxidation of GSH 43. Thus, incorporating Ce6 into phospholipid nanoparticles does not influence its ability to generate ROS under light. Moreover, after irradiation, not only Ce6 itself, but also the products of peroxidation of Ce6–PC and lipoprotein phospholipids become the source of ROS. This effect may increase photocytotoxicity of the Ce6–PC. Such an effect of increasing the photocytotoxicity of liposomal forms of Ce6 derivatives has been described 44.

### Comparative study of Ce6 accumulation in tumor tissue and in the skin of mice with an inoculated Lewis tumor

**Figure 5:**
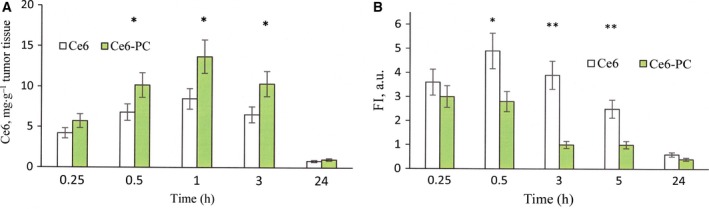
Ce6 accumulation in tumor tissue (A) and in the skin (B) after i.v. administration to mice with inoculated Lewis tumor in the form of free Ce6 and within phospholipid nanoparticles (Ce6–PC). (A) mass spectrometry study of the kinetics of Ce6 accumulation in the tumor tissue; (B) dynamics of alteration of Ce6 fluorescence in the skin of experimental animals. FI, fluorescence intensity. *P <* *0.05 (*n *=* *3); **P <* *0.01 (*n *=* *3).

The pharmacokinetics of drugs equipped with a delivery system changes substantially. We investigated the kinetics of Ce6 accumulation in tumor tissue and in the skin of experimental animals after intravenous administration of Ce6 and Ce6–PC by mass spectrometry and fluorescence. The results of the mass‐spectrometric determination of Ce6 content in the tumor tissue are presented in Fig. 5A. The study has shown that administering Ce6–PC alters the kinetics of PS accumulation in tumors when compared with administration of free Ce6. For example, the amount of Ce6 registered in tumors 1 h after Ce6–PC administration almost doubled (8 *vs* 14 μg·g^−1^ tumor tissue). The difference was maintained for up to 10 h after administration.

In clinical and preclinical studies, fluorimetry is used to determine the PS accumulation in a tissues. This non‐invasive method is based on the fact that the fluorescence intensity of an object depends on the PS content. Figure 5B illustrates the fluorescence of the skin samples of the experimental animals with time after i.v. administration of study drugs. These results reveal that the Ce6 content in the skin after its PS administration in the form of Ce6–PC is much lower and it was eliminated up to five times faster (about 1.5 *vs* 5 h) than in the case of Ce6 administration. This means that the phototoxicity period associated with Ce6–PC administration is significantly shorter.

## Conclusions

The resulting composition of the di‐*N*‐methylglucamine salt of Ce6 and soya phospholipids is presented as an ultrafine emulsion with particle size of 18.4 ± 2.5 nm and zeta potential of −34.6 ± 3.0 mV, and with at least 95% of Ce6 incorporated in the phospholipid nanoparticles. Ce6 incorporation in the phospholipid nanoparticles as a drug delivery system causes a bathochromic shift of Q‐band toward the long‐wave region of the spectrum, increases the intensity of its fluorescence, and has no effect on its ability to generate ROS under irradiation.

Intravenous administration of Ce6 embedded in phospholipid nanoparticles enhances Ce6 accumulation in the tumor tissue. Furthermore, PS accumulates in the skin to a lesser degree and is excreted much faster: its rate of elimination from the skin is up to five times higher. Therefore, the observed alterations of physico‐chemical and biological properties of Ce6 incorporated in the phospholipid nanoparticles should lead to an increase in PDT efficacy and reduce phototoxicity.

## Author contributions

VNP and OMI conceived and designed the project; LVK acquired and analyzed the data; VNP and NVM analyzed and interpreted the data; NVM and OMI wrote the paper.
